# The Validity of the SQoL-18 in Patients with Bipolar and Depressive Disorders: A Psychometric Study from the PREMIUM Project

**DOI:** 10.3390/jcm11030743

**Published:** 2022-01-29

**Authors:** Laurent Boyer, Sara Fernandes, Melanie Faugere, Raphaelle Richieri, Pascal Auquier, Guillaume Fond, Christophe Lancon

**Affiliations:** 1 CEReSS-Health Service Research and Quality of Life Center, Aix-Marseille University, 13005 Marseille, France; sfernandes.sara@gmail.com (S.F.); melanie.faugere@ap-hm.fr (M.F.); pascal.auquier@univ-amu.fr (P.A.); guillaume.fond@gmail.com (G.F.); christophe.lancon@ap-hm.fr (C.L.); 2 Psychiatric Department, Assistance Publique–Hôpitaux de Marseille, 13005 Marseille, France; RaphaelleMarie.RICHIERI@ap-hm.fr

**Keywords:** quality of life, psychiatry, mental health, health services research, bipolar and depressive disorders, bipolar disorders, depressive disorders, schizophrenia

## Abstract

The S-QoL 18 is a self-administered questionnaire that assesses quality of life (QoL) among individuals with schizophrenia. This study aims to validate the S-QoL 18 in bipolar and depressive disorders for a more widespread use in psychiatric settings. This study was conducted in a non-selected sample of individuals with bipolar and depressive disorders in the day hospital of a regional psychiatric academic hospital. Two-hundred and seventy-two stable outpatients with bipolar (*n* = 73) and recurrent and persistent depressive (*n* = 199) disorders were recruited over a 12 month-period. The S-QoL 18 was tested for construct validity, reliability, and external validity. The eight-factor structure of the S-QoL 18 was confirmed by confirmatory factor analysis (RMSEA = 0.075 (0.064–0.086), CFI = 0.972, TLI = 0.961). Internal consistency and reliability were satisfactory. External validity was confirmed via correlations between S-QoL 18 dimension scores, symptomatology, and functioning. The percentage of missing data for the eight dimensions did not exceed 5%. INFIT statistics were ranged from 0.7 to 1.2, ensuring that all items of the scale measured the same QoL concept. In conclusion, the S-QoL 18 appears to be a valid and reliable instrument for measuring QoL in patients with bipolar and depressive disorders. The S-QoL 18 may be used by healthcare professionals in clinical settings to accurately assess QoL in individuals with bipolar and depressive disorders, as well as in schizophrenia.

## 1. Introduction

Mental disorders affect, on average, one in five adults [1]; are leading causes of disability worldwide [2]; and are associated with premature mortality and excess costs [3]. Poor quality has been reported in the diagnosis, treatment, and follow-up of patients with mental disorders, such as schizophrenia and bipolar and depressive disorders [4,5,6]. Quality measurement is fundamental for improving the quality of mental health care in schizophrenia and bipolar and depressive disorders, and identifying where changes are needed.

Patients’ views are now considered to be a key measure of quality of care and health [7]. In particular, quality of life (QoL) is a patient-reported outcome measure (PROMs) that captures a person’s perception of health, and includes important issues (e.g., personal well-being, social interaction, physical health) that are not assessed in traditional measures, such as symptom severity and functioning [8,9,10]. The S-QoL 18 is a widely-used self-administered QoL questionnaire for individuals with schizophrenia [11,12]. The items were developed exclusively on patients’ points of view, and generated from interviews. Interviews with patients are commonly considered as the best method to capture the patient’s perceptions [13]. The S-QoL 18 has also satisfactory psychometric properties in homeless individuals with bipolar disorders [14]. The extent to which the S-QoL 18 remains relevant and valid for bipolar and depressive disorders, including bipolar and recurrent and persistent depressive disorders, is an important issue for widespread use in psychiatric settings that has been insufficiently examined. To date, several QoL questionnaires can be used in bipolar and depressive disorders, but they present several limitations. Generic instruments (e.g., SF-36, WHOQOL) are generally used to compare QoL across different populations, but they lack sensitivity for detecting and quantifying small changes [15]. Disease-specific instruments focus on particular health problems, and are more sensitive to changes, such as the QoL.BD for bipolar disorders, but, to our knowledge, there is no validation of the QoL.BD in depressive disorders [16]. This study aims to validate the S-QoL 18 in bipolar and depressive disorders. This work is a part of the Patient-Reported Experience Measure for Improving qUality of care in Mental health (PREMIUM) project that intends to develop a set of PROMs and patient-reported experience measures (PREMs) to improve the quality of mental health care for adult patients with mental disorders based on modern testing methods [4,17,18].

## 2. Population and Methods

### 2.1. Study Population

All patients have been recruited over a 12 month-period (2019) in the day hospital (stable outpatients) of the regional psychiatric academic hospital (AP-HM, Assistance Publique–Hôpitaux de Marseille, Marseille, France). The patients were referred from the whole Provence-Alpes-Côte-d’Azur region (South-East of France) by their general practitioner or a psychiatrist, who subsequently received a detailed evaluation report with suggestions for personalized interventions. The study was carried out in accordance with ethical principles for medical research involving humans (the seventh revision of the Declaration of Helsinki). The assessment protocol was approved by the relevant ethical review committee (CPP-Sud Méditerranée V, Nice, France; 12 November 2014, n° 2014-A01152-45).

The inclusion criteria were: age ≥18 years, DSM-5 criteria for a diagnosis of bipolar or recurrent or persistent major depressive disorders using Structured Clinical Interview (SCID) [19,20], partial or total remission (defined by absence of acute mood episode for at least 8 weeks using SCID), current ongoing background regimen (mood stabilizers and/or antidepressants), written informed consent, and speaking/reading French.

### 2.2. Data Collection

All patients were interviewed by a psychiatrist using the structured interview for mental disorders to confirm diagnosis of bipolar or recurrent or persistent depressive disorders.

The S-QoL 18 is a self-administered and multidimensional QoL questionnaire that was initially developed for individuals with schizophrenia, including 18 items describing 8 dimensions: Psychological Well-being (PsW), Self-Esteem (SE), Family Relationships (RFa), Relationships with Friends (RFr), Resilience (RE), Physical Well-being (PhW), Autonomy (AU), and Sentimental Life (SL). It also includes a total score (Index) [11]. The eight dimensions and the index score range from 0 to 100; higher scores indicate better QoL. The S-QoL 18 is a French questionnaire available in English and Spanish after a forward-backward translation process, available on request from Mapi Trust Research (https://eprovide.mapi-trust.org/instruments/schizophrenia-quality-of-life-questionnaire-short-form-clinical-practice. Accessed on 27 January 2022).

Socio-demographic and clinical data were collected: sex, age, marital status (single, yes/no), educational level (<12/≥12 years), employment (yes/no), severity of symptoms using the Clinical Global Impression (CGI, score ranging from 1 (normal) to 7 (among the most extremely ill patients)) [21], the Hamilton Depression Rating hetero-rated scale (score ranging from 0 to 54, with higher scores indicating more severe depressive symptomatology) [22], the Young Mania Rating Scale (YMRS, score ranging from 0 to 60, with higher scores indicating more severe mania) [23], adherence to treatment using the Medication Adherence Rating Scale (MARS, score ranging from 0 to 10, with a higher score indicating better medication adherence) [24], functioning using the Global Assessment Functioning scale (GAF, score ranging from 1 (severely impaired) to 100 (extremely high functioning)) [25], and the SF-36 [26]. The SF-36 is a self-administered questionnaire consisting of 36 items describing 8 dimensions: physical functioning (PF); social functioning (SF); role—physical problems (RPP); role—emotional problems (REP); mental health (MH); vitality (VIT); bodily pain (BP); and general health (GH). It also included two composite scores: SF-36 physical (PCS) and mental (MCS) composite scores. Each dimension and composite score is scored within a range from 0 (low functioning level) to 100 (high functioning level).

### 2.3. Statistical Analysis

Descriptive statistics of the sample included frequencies and percentages of categorical variables, and the means and standard deviations of continuous variables. Floor and ceiling effects were reported assessing the homogeneous repartition of the response distribution. The structure of the S-QoL 18 was explored using confirmatory factor analysis, and the following indices were used to assess the goodness of fit of the model to the data, with acceptable fit defined as the root mean square error of approximation (RMSEA) ≤ 0.08, and the comparative fit index (CFI) and the Tucker–Lewis index (TLI) ≥ 0.95 [27]. The unidimensionality of each dimension was assessed using a Rasch analysis by using the partial credit model (PCM). The goodness-of-fit was assessed by computing the INFIT statistics; a value of INFIT between 0.7 and 1.3 ensures that all items of the scale tend to measure the same concept. Differential item functioning (DIF) analyses were performed to see whether all items of the S-QoL 18 behave in the same way across different subgroups, identified by sex (man vs. woman), age (mean age: <46/≥46 years), educational level (<12/≥12 years), and diagnosis (bipolar/major depressive disorders) [28]. If an overall DIF was detected at the level of *p* < 0.01, the magnitude was assessed according to Zumbo’s DIF classification by computing the pseudo R² change (ΔR²): negligible if ΔR² < 0.13, moderate if 0.13 < ΔR² < 0.26, and large if ΔR² > 0.26 [29].

Additionally, item-internal consistency was assessed by correlating each item with its scale (corrected for overlap) using Pearson’s coefficient (a coefficient of at least 0.4 was expected for supporting item-internal consistency [30]); item discriminant validity was assessed by determining the extent to which items correlate more highly with the dimensions they are hypothesized to represent than with the others [31]. For each dimension scale, internal consistency reliability was assessed using Cronbach’s alpha coefficient (a coefficient of at least 0.7 was expected for each scale) [32,33].

Finally, to explore external validity, Pearson’s correlation coefficients were used to investigate relationships between dimensions of the S-QoL 18 and CGI, Hamilton scale, YMRS, MARS, GAF, and SF-36. Discriminant validity was also examined by testing the association of the S-QoL 18 scores with sociodemographic (age, sex, educational level, marital status, and employment status) and clinical (diagnosis) characteristics using *t* tests and Pearson’s correlation coefficients. Several hypotheses were formulated: the S-QoL 18 (1) should be lower in female, low education level, single, and unemployed patients [34]; (2) should be negatively correlated with the severity of depressive symptoms (CGI, Hamilton) [35,36]; (3) should be negatively correlated with the severity of manic symptoms (YMRS) [37]; (4) should be positively, but moderately, correlated with adherence to treatment (MARS) [38] and functioning (GAF and SF-36) [34,39].

The acceptability of measuring QoL was tested using the percentage of missing values for the S-QoL 18.

## 3. Results

### 3.1. Sample Characteristics

Two-hundred and seventy-two patients with bipolar (*n* = 73) and major depressive (*n* = 199) disorders were recruited. The characteristics of patients are presented in Table 1. Patients had moderate residual depressive symptoms (mean Hamilton score = 15.5) without manic symptoms (mean YMRS score = 2.1). Functioning scores were particularly low on the mental dimensions of the SF-36 (MCS = 27.6).

### 3.2. Construct Validity, Internal Structural Validity, and Reliability

All of the details are provided in Table 2. QoL scores were low for all dimensions (<50 except for family relationships and autonomy). The three lowest dimensions were self-esteem, physical well-being, and sentimental life. The eight-factor structure of the S-QoL 18 was confirmed by confirmatory factor analysis (RMSEA = 0.075 (0.064–0.086), CFI = 0.972, TLI = 0.961). The overall scalability was satisfactory. All of the items showed a good fit for the Rasch model in each dimension, and none of the items had a statistical INFIT outside the range of acceptability. Item internal consistency was satisfactory for all dimensions, and each item achieved the 0.40 standard (ranging from 0.49 to 0.82), except for the psychological well-being dimension (acceptable min with 0.35). The correlation of each item with its contributive dimension was higher than that with the other dimensions for seven of the eight dimensions (item discriminant validity). Cronbach’s alpha coefficients were higher than 0.70 (from 0.73 to 0.89), indicating satisfactory reliability, with one exception of psychological well-being dimension, which was higher than 0.60. Floor and ceiling effects were less than 20%, except for one dimension (sentimental life). Items did not show significant overall DIF by sex (man vs. woman), age (mean age: <46/≥46 years), educational level (<12/≥12 years), and diagnosis (bipolar/major depressive disorders), except for item 8, which was significant, but with negligible magnitude (Appendix A).

### 3.3. External Validity of the SQOL-18 (Index)

All of the details are provided in Table 3. The S-QoL 18 did not reveal any statistically significant association with age and sex (but we noted a statistical trend for lower quality of life in females compared to males, *p* = 0.096). The S-QoL 18 was lower in single, unemployed, and low educational level (statistical trend: *p* = 0.055) patients.

As expected, the S-QoL 18 index was significantly, but moderately, correlated with symptom severity (CGI, Hamilton), adherence (MARS), and functioning (GAF and SF-36). In contrast, YMRS was not significantly associated with the S-QoL.

Complementary analyses on the correlations between the S-QoL 18 dimensions and the SF-36 dimensions can be found in Appendix B.

The items of the S-QoL 18 are presented in Appendix C.

## 4. Discussion

In this study, we demonstrated the construct validity, reliability, external validity, and acceptability of the S-QoL 18 in individuals with bipolar and depressive disorders, similarly with our previous works on schizophrenia [11,12,40,41]. The S-QoL 18 presents interesting characteristics for widespread use in clinical settings.

QoL is multidimensional concept that can be more easily measured by psychometrically valid and reliable multidimensional questionnaires than with a simple question. A large number of QoL instruments have already been validated and used in patients suffering from bipolar and depressive disorders [16,42,43], including generic questionnaires lacking the sensitivity to change, and specific questionnaires, such as the QoL-BD [16], capturing the particular priorities of individuals living with bipolar disorders. The S-QoL 18 was initially developed for individuals with schizophrenia, but our findings confirmed its interest in bipolar and depressive disorders. The S-QoL 18 could therefore be relevant as a useful “intermediate” questionnaire between generic and specific tools, which could be extended to several mental disorders. Among its advantages, the S-QoL 18 is one of the shortest multidimensional instruments in QoL measures. Some S-QoL 18 dimensions are close to those referred to, in both the literature and existing tools, as psychological and physical well-being. However, some dimensions, such as resilience, autonomy, relationships with family and friends, and sentimental life, are rarely measured, whereas the social dimension is strongly impacted in bipolar and depressive disorders [44,45]. Contrary to North American or English questionnaires, such as the BD-QoL, specific aspects, such as availability of money or quality of accommodation, were not explored. This difference may reflect health the care specificities of the French health system, with its universal coverage. As such, our questionnaire may be of particular interest in health care systems characterized by universal access to care. Another cultural difference between our French questionnaire and North American or English equivalents is that the former lacks a dimension that measures spirituality and religious beliefs. Last, the S-QoL also exists in a computerized adaptive testing (CAT) for patients with schizophrenia [46]. Tailored for patient characteristics and significantly shorter than the paper-based version, the SQoL-CAT may improve the feasibility of assessing QoL in clinical practice.

The S-QoL 18 demonstrated satisfactory psychometric properties. In particular, the structure of the S-QoL 18 was confirmed with acceptable fit (RMSEA, CFI, and TLI) [27]. The unidimensionality of each dimension and the goodness-of-fit were also confirmed using Rasch analysis and INFIT statistics. Importantly, the differential item functioning analyses confirmed that all items of the S-QoL 18 behave in the same way regardless of the characteristics of patients. This property is rarely studied in other questionnaires, and we can thus speculate that responses to S-QoL 18 are comparable according to the responders’ characteristics, especially for bipolar and recurrent or persistent depressive disorders. Internal consistency reliability of the eight dimensions has been shown to be acceptable. The percentage of missing data for the eight dimensions did not exceed 5%, confirming a satisfactory acceptability. External validity, explored by the use of socio-demographic characteristics and established psychiatric and functioning measures, globally confirmed our assumptions. The S-QoL 18 was lower in female (statistical trend in our study), low education level (statistical trend in our study), single, and unemployed patients [34]. Robb et al. reported gender differences in QoL scores; women possessed numerically lower scores in different dimensions except for mental health, with significant differences in the domains of pain and physical health [47]. Educational level, social disadvantage, and unemployment were reported to be at greater risk for improvement in quality of life and functioning [34,48]. Marital status was also associated with quality of life in patients with recurrent or persistent depressive disorder [49]. The S-QoL 18 was negatively correlated with the severity of depressive symptoms in accordance with previous works [35,36]. On the opposite, the S-QoL 18 was not associated with the severity of manic symptoms [37]. This is probably due to the absence of manic symptoms in our stabilized population. Future work should explore this association in a sample with patients with residual manic symptoms, and differentiating different types of manic symptoms (e.g., tension/aggressiveness-induced impaired QoL versus euphoria/hypersociability, which may induce an increased self-reported QoL). As expected, the S-QoL 18 was lower in patients with lower adherence to treatment [38,50]. The S-QoL 18 was negatively and moderately associated with functioning (GAF and SF-36) [34,39], especially for dimensions such as resilience, autonomy, relationships with family and friends, and sentimental life, confirming the relevance of S-QoL 18 as a complement to more traditional and objective evaluation and examination.

### Limitations and Perspectives

Several limitations in the process of validation of the SQoL 18 must be noted. The sample may not be representative of the entire population of bipolar and depressive disorders patients, and our findings should be confirmed in larger and multicenter samples. The participants were recruited with a whole year and seasonality may have influenced mood and therefore QoL results [51,52]. Criterion validity is considered present when the measurement predicts an external criterion based on a gold standard. In the case of QoL, there is no gold standard, and the instrument is considered valid if it consistently fits a series of related concepts. In our study, we made comparisons with other measures of functioning (GAF and SF-36), symptomatology (CGI, Hamilton, YMRS), and medication adherence (MARS). Although this choice can be debatable, it can be assumed that our assumptions based on the relationships between the S-QoL 18 and these scales are both reasonable and pragmatic. The sensitivity to change was not explored, and should be studied in future works. This property is of particular interest for the follow-up of patients in clinical practice.

## 5. Conclusions

The S-QoL 18 appears to be a valid and reliable instrument for measuring QoL in patients with bipolar and depressive disorders. The S-QoL 18 may be used by healthcare professionals in psychiatric settings to accurately assess QoL in individuals with bipolar and depressive disorders, as well as in schizophrenia.

## Figures and Tables

**Table 1:** Characteristics of patients (*n* = 272).

Variables	N(%) or Mean (Standard Deviation)	Whole Sample (*n* = 272)	Patients with Bipolar Disorders (*n* = 73)	Patients with Depressive Disorders (*n* = 199)	*p*-Value
Sex	Men	118 (43.4)	27 (37.0)	91 (45.7)	
	Women	154 (56.6)	46 (63.0)	108 (54.3)	0.197
Marital status (single)	No	121 (45.0)	26 (36.1)	95 (48.2)	
	Yes	148 (55.0)	46 (63.9)	102 (51.8)	0.077
Educational level	<12 years	88 (34.9)	19 (30.2)	69 (36.5)	
	≥12 years	164 (65.1)	44 (69.8)	120 (63.5)	0.360
Employment	No	207 (77.2)	57 (80.3)	150 (76.1)	
	Yes	61 (22.8)	14 (19.7)	47 (23.9)	0.476
Diagnosis	Bipolar disorders	73 (26.8)	-	-	
	Type 1	30			
	Type 2	33			
	Missing data	10			
	Recurrent and persistent depressive disorders	199 (73.2)			
Age		46.2 (15.5)	44.5 (15.0)	46.9 (15.6)	0.259
CGI score		4.1 (1.3)	4.1 (1.3)	4.1 (1.3)	0.978
Hamilton score		15.5 (8.5)	13.7 (9.7)	16.0 (8.0)	0.185
YMRS score		2.1 (4.7)	4.8 (8.1)	1.2 (2.3)	0.015
MARS score		6.1 (2.2)	6.1 (2.3)	6.1 (2.2)	0.914
GAF score		57.8 (15.8)	57.7 (16.1)	57.9 (15.7)	0.949
SF-36 score	PF	69.5 (26.1)	67.1 (28.0)	70.2 (25.6)	0.475
	SF	37.7 (23.6)	34.9 (26.8)	38.6 (22.5)	0.347
	RPP	36.8 (40.5)	39.1 (42.8)	36.1 (39.8)	0.658
	REP	21.7 (34.6)	23.6 (37.0)	21.0 (33.9)	0.654
	MH	37.9 (21.4)	37.7 (24.4)	38.0 (20.4)	0.919
	VIT	32.7 (20.2)	35.9 (25.9)	31.7 (18.0)	0.296
	BP	56.2 (27.9)	58.8 (26.7)	55.3 (28.3)	0.453
	GH	43.4 (19.1)	46.3 (21.2)	42.5 (18.3)	0.233
	MCS	27.6 (11.4)	27.9 (13.4)	27.5 (10.8)	0.851
	PCS	45.8 (10.2)	46.1 (11.2)	45.7 (9.9)	0.781

Notes: CGI, Clinical Global Inventory Scale; YMRS, Young Mania Rating Scale; MARS, Medication Adherence Rating Scale; GAF, Global Assessment of Functioning Scale; PF, physical functioning; SF, social functioning; RPP, role—physical problems; REP, role—emotional problems; MH, mental health; VIT, vitality; BP, bodily pain; GH, general health; PCS/MCS, SF-36 physical and mental composite scores.

**Table 2:** Dimension characteristics of the S-QoL 18 (*n* = 272).

Dimension and Index (Number of Items)	Mean (Standard Deviation)	Missing Data %	Item Internal Consistency Min–-Max	Item Discriminant Validity Min–Max	Floor Effect %	Ceiling Effect %	Alpha	INFIT Min–Max
PsW (3) (0–100)	43.9 (26.8)	0	0.35–0.50	0.16–0.55	7.7	2.9	0.63	0.88–1.11
SE (2) (0–100)	33.7 (28.0)	0.7	0.60	0.19–0.60	19.5	1.8	0.75	0.68–0.71
RFa (2) (0–100)	58.9 (32.4)	2.9	0.82	0.03–0.46	11.8	16.5	0.90	1.18–1.19
RFr (2) (0–100)	49.2 (30.6)	3.3	0.58	0.09–0.39	12.1	6.3	0.73	1.03–1.06
RE (3) (0–100)	46.2 (29.3)	0	0.49–0.63	0.03–0.48	9.9	5.1	0.76	0.95–1.16
PhW (2) (0–100)	32.3 (28.3)	0.4	0.66	0.14–0.51	25.0	2.6	0.80	0.81–1.00
AU (2) (0–100)	52.4 (29.7)	0.4	0.80	0.17–0.51	11.4	6.3	0.89	0.75–0.81
SL (2) (0–100)	35.1 (33.96)	4.4	0.78	0.12–0.42	35.7	6.3	0.88	0.91–1.04
Index (18) (0–100)	43.8 (19.5)	8.1	NA	NA	NA	NA	0.88	NA

Notes: NA, not applicable; PsW, psychological well-being; SE, self-esteem; RFa, family relationships; RFr, relationships with friends; RE, resilience; PhW, physical well-being; AU, autonomy; SL, sentimental life.

**Table 3:** External validity of the SQoL-18 (*n* = 272).

Variables		Correlation Coefficient (*r*)	Mean (Standard Deviation)	*p*-Value
Sex	Men	-	49.16 (18.15)	0.096
	Women		45.10 (19.90)	
Marital status (single)	No	-	50.09 (18.60)	**0.008**
	Yes		43.64 (19.08)	
Educational level	<12 years	-	43.88 (16.65)	0.055
	≥12 years		48.71 (20.52)	
Employment	No	-	44.51 (19.32)	**<0.001**
	Yes		54.96 (17.12)	
Diagnosis	Bipolar disorders		47.04 (20.07)	0.961
	Major depressive disorder		46.90 (18.97)	
Age		−0.038	-	0.544
CGI		−0.508	-	**<0.001**
Hamilton		−0.521	-	**<0.001**
YMRS		−0.022	-	0.797
MARS		0.218	-	**0.001**
GAF		0.502	-	**<0.001**
SF-36	PF	0.281	-	**<0.001**
	SF	0.581	-	**<0.001**
	RPP	0.411	-	**<0.001**
	REP	0.470	-	**<0.001**
	MH	0.587	-	**<0.001**
	VIT	0.547	-	**<0.001**
	BP	0.290	-	**<0.001**
	GH	0.558	-	**<0.001**
	MCS	0.610		**<0.001**
	PCS	0.270		**<0.001**

Notes: CGI, Clinical Global Inventory Scale; MARS, Medication Adherence Rating Scale; GAF, Global Assessment of Functioning Scale; PF, physical functioning; SF, social functioning; RPP, role—physical problems; REP, role—emotional problems; MH, mental health; VIT, vitality; BP, bodily pain; GH, general health; PCS/MCS, SF-36 physical and mental composite scores. In bold, *p*-values < 0.05.

## Data Availability

The data are available on demand from the PREMIUM Scientific Committee.

## Appendix A

**Table A1:** DIF Results.

Items	Sex		Age		Diagnosis		Educational Level	
	*p* Value	ΔR²	*p* Value	ΔR²	*p* Value	ΔR²	*p* Value	ΔR²
1	0.879	-	0.002	-	0.727	-	0.197	-
2	0.673	-	0.515	-	0.939	-	0.594	-
3	0.018	-	0.197	-	0.550	-	0.946	-
4	0.376	-	0.123	-	0.121	-	0.189	-
5	0.284	-	0.883	-	0.909	-	0.348	-
6	0.990	-	0.359	-	0.330	-	0.269	-
7	0.236	-	0.896	-	0.345	-	0.561	-
8	0.003	<0.13	0.266	-	0.356	-	0.861	-
9	0.124	-	0.722	-	0.225	-	0.903	-
10	0.367	-	0.340	-	0.387	-	0.999	-
11	0.105	-	0.456	-	0.658	-	0.913	-
12	0.214	-	0.857	-	0.788	-	0.185	-
13	0.304	-	0.766	-	0.078	-	0.011	-
14	0.298	-	0.772	-	0.584	-	0.549	-
15	0.528	-	0.581	-	0.423	-	0.962	-
16	0.701	-	0.583	-	0.820	-	0.245	-
17	0.824	-	0.318	-	0.951	-	0.206	-
18	0.766	-	0.505	-	0.086	-	0.325	-

Notes: ΔR²: DIF magnitude: negligible (ΔR² < 0.13), moderate (0.13 ≤ ΔR² ≥ 0.26), or large (ΔR² ≥ 0.26).

## Appendix B

**Table A2:** Correlations between the S-QoL 18 Dimensions and the SF-36 Dimensions.

S-QoL 18\SF-36	PF	SF	RPP	REP	MH	VIT	BP	GH	MCS	PCS
PsW	**0.174 ***	**0.572 ****	**0.316 ****	**0.474 ****	**0.606 ****	**0.394 ****	**0.146 ***	**0.387 ****	**0.635 ****	0.091
SE	**0.199 ****	**0.565 ****	**0.297 ****	**0.451 ****	**0.669 ****	**0.559 ****	**0.239 ****	**0.480 ****	**0.657 ****	0.121
Rfa	0.000	**0.238 ****	**0.195 ****	**0.201 ****	**0.212 ****	**0.172 ***	**0.152 ***	**0.186 ***	**0.236 ****	0.094
RFr	**0.165 ***	**0.375 ****	**0.270 ****	**0.362 ****	**0.395 ****	**0.395 ****	**0.280 ****	**0.331 ****	**0.409 ****	**0.193 ****
RE	**0.188 ****	**0.388 ****	**0.208 ****	**0.281 ****	**0.353 ****	**0.412 ****	0.084	**0.331 ****	**0.389 ****	0.103
PhW	**0.442 ****	**0.404 ****	**0.394 ****	**0.349 ****	**0.456 ****	**0.632 ****	**0.377 ****	**0.597 ****	**0.404 ****	**0.440 ****
AU	**0.243 ****	**0.496 ****	**0.247 ****	**0.353 ****	**0.401 ****	**0.395 ****	**0.255 ****	**0.446 ****	**0.424 ****	**0.206 ****
SL	0.099	**0.393 ****	**0.149 ***	**0.257 ****	**0.390 ****	**0.216 ****	0.075	**0.355 ****	**0.387 ****	0.033

Notes: S-QoL 18: PsW, psychological well-being; SE, self-esteem; RFa, family relationships; RFr, relationships with friends; RE, resilience; PhW, physical well-being; AU, autonomy; SL, sentimental life. SF-36: PF, physical functioning; SF, social functioning; RPP, role—physical problems; REP, role—emotional problems; MH, mental health; VIT, vitality; BP, bodily pain; GH, general health. In bold: statistically significant, * *p* < 0.05, ** *p* < 0.01.

## Appendix C. List of S-QoL 18 Items (Dimension)

At the present time,

1. I am confident in life (Self-esteem)
2. I fight to succeed in my life (Resilience)
3. I am able to plan for my professional or personal future (Resilience)
4. I am in a good mood. I am at ease with myself (Self-esteem)
5. I feel free to make decisions (Autonomy)
6. I feel free to act (Autonomy)
7. I make efforts to work (Resilience)
8. I am in good physical shape (Physical well-being)
9. I am full of energy (Physical well-being)
10. I am helped and supported by my family (Family relationships)
11. My family pays attention to me (Family relationships)
12. I am helped and supported by my friends or my relatives (Relationships with friends)
13. I have friends (Relationships with friends)
14. I am satisfied with my love life (Sentimental life)
15. I am able to achieve my sentimental projects (Sentimental life)
16. I have difficulty concentrating or thinking straight (Psychological wellbeing)
17. I feel cut off from the outside world (Psychological wellbeing)
18. I have difficulty expressing my feelings (Psychological wellbeing)
